# Retention, Bacterial Adhesion, and Biofilm Formation between Anionic and Zwitterionic Bandage Contact Lenses in Healthy Dogs: A Pilot Study

**DOI:** 10.3390/vetsci8100238

**Published:** 2021-10-18

**Authors:** Mizuki Kita, Kazutaka Kanai, Hisaya K. Ono, Yuya Otaka, Daiki Okada, Noriaki Nagai, Rina Kudo, Yohei Yamashita, Shiori Hino, Toru Matsunaga, Kazuki Tajima

**Affiliations:** 1 Department of Small Animal Internal Medicine II, School of Veterinary Medicine, Kitasato University, 35-1 Higashi 23 ban-cho, Towada, Aomori 034-8628, Japan; dv18003@st.kitasato-u.ac.jp (M.K.); dv20001@st.kitasato-u.ac.jp (Y.O.); dv21002@st.kitasato-u.ac.jp (D.O.); vm16502@st.kitasato-u.ac.jp (R.K.); ebisu2101@yahoo.co.jp (Y.Y.); tajima@vmas.kitasato-u.ac.jp (K.T.); 2 Department of Zoonoses, School of Veterinary Medicine, Kitasato University, 35-1 Higashi 23 ban-cho, Towada, Aomori 034-8628, Japan; hisaono@vmas.kitasato-u.ac.jp; 3 Faculty of Pharmacy, Kindai University, 3-4-1 Kowakae, Higashi-Osaka, Osaka 577-8502, Japan; nagai_n@phar.kindai.ac.jp; 4 SEED Co., Ltd., 2-40-2 Hongo, Bunkyo-ku, Tokyo 113-8402, Japan; s_hino@seed.co.jp (S.H.); toru_matsunaga@seed.co.jp (T.M.)

**Keywords:** bacterial adhesion, bandage contact lens, biofilm formation, contact lens retention, dogs

## Abstract

This study aimed to compare the in vitro and in vivo retention, bacterial adhesion, and biofilm formation between anionic and zwitterionic bandage contact lenses (BCLs) in healthy canines. BCL retention and tolerance were evaluated in 10 healthy canines via a single-masked, crossover study for 7 days. To compare in vitro bacterial adhesion and biofilm formation, four *Staphylococcus* strains were incubated with the BCLs at 37 °C for 2 or 24 h, and the bacterial colony forming units (CFUs) adhering to the BCLs were counted. Next, to compare in vivo bacterial adhesion, the CFUs of bacteria adhering to the BCLs worn by canines for 24 h were counted. Anionic lenses significantly retained and reduced in vitro bacterial adhesion than in the zwitterionic lenses. However, the amount of in vitro biofilm formation was more likely to be higher on anionic lenses than on zwitterionic lenses. In vivo bacterial adhesion was not significantly different between the two types of BCLs. Nevertheless, both BCLs were well-tolerated by the canines; thus, their short-term use in dogs can be recommended as safe.

## 1. Introduction

In the veterinary field, bandage contact lenses (BCLs) are used to treat corneal disease, protect the cornea, and relieve pain [1,2,3,4]. In particular, the use of BCL can notably reduce the wound healing time in canines with spontaneous chronic corneal epithelial defects (SCCEDs) [1,3,4]. However, BCLs must be retained to reduce the healing time. If the used BCL is lost from the corneal surface within 7 days, it does not shorten the corneal healing time in SCCEDs [1].

Retention is dependent on how the BCL fits in the cornea. In the field of human ophthalmology, anterior corneal curvature is routinely measured using a keratometer while fitting BCLs [5,6,7]. However, in veterinary ophthalmology, keratometry is not commonly used in selecting BCL sizes [1,2,3,4,8,9]. In addition, there are considerable variations in corneal curvature among canine breeds and weights. Most veterinary ophthalmologists are likely to stock no more than a few BCLs of different types and sizes [9,10]. Thus, BCLs having an appropriate base curve (BC) for the canine corneal curvature may not always be applied. Thus, BCLs that fit a wider range of corneal curvatures should be developed.

During BCL usage, microbial adhesion and biofilm formation represent serious threats to corneal health. Mature biofilm development is associated with infections such as microbial keratitis, which can induce corneal opacity and melting corneal ulcers, leading to visual impairment [11,12]. When a BCL is placed on the cornea, it takes up proteins and other substances from the tear fluid, creating a suitable environment for microorganism growth [13,14,15]. Once planktonic bacteria adhere to the BCL, they form microcolonies or biofilms [13,14,15,16,17]. Biofilm is a complex of microbial communities adhered to a surface and contained within exopolysaccharides, which protect bacteria from host defenses and/or antibiotics [13,16,17].

Protein absorption and bacterial adhesion are dependent on the material characteristics of BCL, of one is the surface charge. BCLs are classified as anionic, zwitterionic, and nonionic according to their surface charge. Anionic lenses have negative ionic properties. Zwitterionic lenses possess both positive and negative properties, with an overall neutral charge. Nonionic lenes have no ionic properties. In general, proteins have a positive charge, and thus have an affinity to negatively charged BCLs [14,18]. In contrast, the surface of nearly all bacteria is negatively charged, including *staphylococcus* spp., which is one of the most common components of canine conjunctival flora and is a pathogen implicated in microbial keratitis [8,19,20,21,22,23,24]. Eighty percent of bacterial corneal ulcers are caused by *Staphylococcus aureus*, *Streptococcus pneumoniae*, and *Pseudomonas* species. The ability of an organism to adhere to the edge or base of a corneal epithelial defect is causative for pathogenicity [25]. Therefore, negatively charged bacteria cannot easily adhere to anionic materials due to electrostatic repulsion [21].

Several studies have investigated BCL retention in canines; however, few have compared differences in retention among BCL materials [1,2,3,4,8,9]. Therefore, this study aimed to compare retention and in vitro and in vivo bacterial adhesion and biofilm formation between two differently charged BCLs with similar BCs, diameters, central thicknesses, and water contents.

## 2. Materials and Methods

### 2.1. Animals

The study population comprised 10 beagles kept for teaching purposes by the School of Veterinary Medicine, Kitasato University. The average age and weight of the animals were 2.4 ± 0.6 years and 11.6 ± 0.8 kg, respectively. Prior to the study, all canines were examined by a board-certified veterinary ophthalmologist. Only dogs with a complete eye examination that was within normal limits were included in the study. The ophthalmic examinations included the Schirmer’s tear Test 1 (Schirmer Tear Production Measuring Strips, AYUMI Pharmaceutical Corporation, Tokyo, Japan), fluorescein staining (FLURORES Ocular Examination Test Paper 0.7 mg, AYUMI Pharmaceutical Corporation, Tokyo, Japan), applanation tonometry (Tono-Pen AVIA Vet, Reichert Inc., NY, the USA) after the administration of 0.4% topical oxybuprocaine (Benoxil, Santen Pharmaceutical Co., Ltd., Osaka, Japan), slit-lamp biomicroscopy (Kowa SL-15, Kowa Co., Ltd., Tokyo, Japan), and indirect ophthalmoscopy (Volk 20D lens, Volk Optical Inc., Mentor, Ohio). The canines were housed in individual cages under controlled environmental conditions (light phase from 7 am to 7 pm and dark phase from 7 p.m. to 7 a.m.). They were cared for in accordance with the guidelines of the Animal Care and Use Committee of Kitasato University.

### 2.2. Contact Lenses

Two types of ionic BCLs were provided by SEED Co., Ltd., Tokyo, Japan. Table 1 shows the differences between the BCLs.

### 2.3. Bacterial Strains

We used four different bacterial strains, two of *S. aureus* reference strains (N315 and RN4220) and two of *S.* *pseudintermedius* isolated from the conjunctival sac of healthy canines. The isolation protocol of *S. pseudintermedius* is described below.

### 2.4. Bacterial Isolation

Two bacterial samples from the ventral conjunctival sac were taken from one eye of each of two healthy beagles using sterilized cotton swabs. The swab samples were suspended in 1 mL of the brain heart infusion broth (Eiken Chemical Co., Ltd., Tokyo, Japan) with 7.5% sodium chloride and were incubated for 48 h at 37 °C. After incubation, each bacterial suspension was inoculated into mannitol salt agar (Eiken Chemical Co., Ltd., Tokyo, Japan) with an egg yolk (MSEY) plate and were incubated for 48 h at 37 °C. Presumptive *S. pseudintermedius* colonies were confirmed with the coagulase test using rabbit plasma (Eiken Chemical Co., Ltd., Tokyo, Japan). Further confirmatory tests for *S. pseudintermedius* were performed on coagulase-positive isolates (multiplex polymerase chain reaction) [26]. Total DNA was extracted from one colony of each sample with MightyPrep for DNA analysis (Takara Bio Inc., Shiga, Japan), according to manufacturer’s instruction.

### 2.5. Selecting the Size of BCL

The BCL sizes were chosen based on the canine’s weight, according to a previous report [10]. For individuals weighing less than 12 kg, BCLs with a BC of 8.9 mm were used. For individuals weighing more than 12 kg, BCLs with a BC of 9.3 mm were used.

### 2.6. Retention

This was a single-masked crossover study. First, the A lens was placed on the cornea of each dog and the B lens on the cornea of the contralateral eye using sterilized soft tip forceps. Caution was taken to ensure that no air bubble was captured between the BCL and the cornea and that the nictitating membrane glided smoothly over the BCL. The BCL, which remained on the corneal surface, was removed after 7 days. We set a withdrawal period of 7 days, without lenses, before the same experiment was conducted by switching the placement of the left and right lenses. The canines were assessed using a slit-lamp biomicroscope 1, 3, 6, and 12 h after BCL placement and every 12 h thereafter to determine whether the BCL was retained. Ocular irritation was recorded during daily checkups. We assessed the animals using semi-quantitative scores for subjective discomfort, ocular discharge, and conjunctival hyperemia, which are indicative of ocular irritation, based on a few previous study (0 = absent, 1 = mild, 2 = moderate, and 3 = severe) [8,27]. Subjective discomfort was evaluated on the basis of reduced palpebral fissure size, blepharospasm, and scratching. After 7 days of observation, the BCL was removed, and fluorescein staining was performed. When the BCL was detached, fluorescein staining was performed. Daily check-ups and fluorescein staining were performed by the same observer, who was unaware of which BCL type was placed on the cornea of which eye in each dog.

### 2.7. In Vitro Bacterial Adhesion

The tryptic soy broth (TSB; Eiken Chemical Co., Ltd., Tokyo, Japan) overnight cultures of four bacteria (described in Section 2.3.) were centrifuged for 10 min at 3000 rpm and washed three times with phosphate-buffered saline (PBS). Then, the bacterial cell suspensions were diluted in the ratio of 3.5:1 in PBS. The sterile BCLs were washed with PBS three times and trephined with an 8-mm disposable biopsy punch (Kai Industries Co., Ltd., Gifu, Japan). Next, each BCL was placed in a 24-well tissue culture plate. Then, 1000 µL of diluted bacterial suspensions were transferred in the wells (2.2 × 10^9^ colony forming units [CFUs] per well for KS201; 2.4 × 10^9^ CFU/well for KS202; 0.6 × 10^9^ CFU/well for N315; 1.3 × 10^9^ CFU/well for RN4220). After incubation of the bacterial suspension for 2 h at 37 °C, each BCL was cautiously removed and washed three times with PBS. Next, each BCL was transferred into a sterile 1.5-mL microtube containing 1000 µL of PBS. Next, the tubes were sonicated for 1 min to separate the cells from BCL. The cell suspensions were serially (10-fold) diluted. Fifty microliters of each cell suspension was plated onto the MSEY plates, and CFUs were counted after incubation at 37 °C for 18 h. The experiment was performed once using six A lenses and six B lenses for each of the four bacteria. The experimental conditions were designed based on previous reports [13,28] and validated in a preliminary study.

### 2.8. In Vitro Biofilm Formation

The TSB overnight culture of each bacterium was diluted with 10^8^:1 TSB containing 0.5% glucose and 3.0% NaCl (approximately 4 × 10^1^ CFU/mL). The 8-mm BCL was washed three times and placed in a 24-well tissue culture plate. Next, the wells were filled with 1000 µL of diluted bacterial suspensions and incubated for 24 h at 37 °C. Each BCL was then cautiously removed and washed three times with PBS and transferred to a sterile 1.5-mL microtube containing 300 µL of 1-mm diameter sterile glass beads in 1000 µL of PBS. Then, the tubes were vortexed for 1.5 min to separate the biofilm matrix cells from the BCL. After vortexing, the cell suspensions were serially (10-fold) diluted. Fifty microliters of each cell suspension was plated onto the MSEY plates, and CFUs were counted after incubation at 37 °C for 18 h. The experiment was performed once using six A lenses and six B lenses for each of the four bacteria. The experimental conditions were designed based on previous reports [13,29,30] and validated in a preliminary study.

### 2.9. In Vivo Bacterial Adhesion

The A or B lenses were used similarly to the experiment, as shown in Section 2.6, in all canines. After 24 h of wearing BCL, the BCL that remained on the corneal surface was removed using sterilized soft tip forceps. Caution was taken to avoid the BCL from coming into contact with the eyelids and eyelashes. Next, it was transferred to a 1.5-mL tube containing 300 µL of glass beads in 1000 µL of PBS. The tubes were then vortexed for 1.5 min. After vortexing, 100 µL of cell suspension was plated onto standard agar plates (Eiken Chemical Co., Ltd., Tokyo, Japan), and the CFUs were counted after incubation. After the 7-day withdrawal period, the same experiment was conducted by switching the left and right lenses. The experimental conditions were designed based on previous reports [30,31] and validated in a preliminary study.

### 2.10. Statistical Analysis

For retention, the time-to-event curves were generated using the Kaplan–Meier method, and the statistical analyses of each BCL were performed using the log-rank test. Further analysis of retention percentage at 168 h (7 days) after wearing the BCL was performed for each BCL using the chi-squared test. For the in vitro and in vivo bacterial adhesion and biofilm formation, the CFU was statistically analyzed among the BCLs based on each bacterial strain using the nonparametric Mann–Whitney U test. All statistical analyses were performed using EZR v. 1.54 (Saitama Medical Center, Jichi Medical University, Saitama, Japan). *p* value of < 0.05 was considered statistically significant.

## 3. Results

### 3.1. Retention

**Figure 1:**
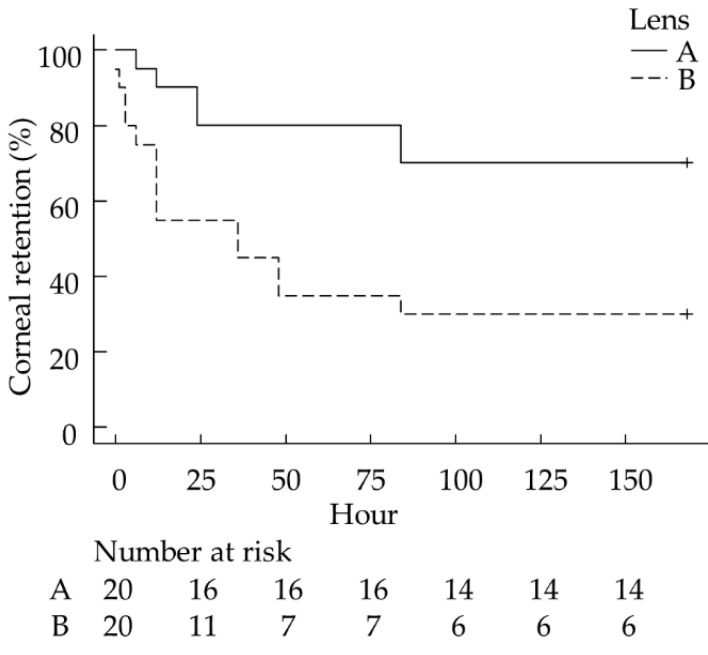
Kaplan–Meier curves for the retention rate of each BCL. The A lens had a significantly higher retention rate than the B lens (*p* = 0.006). BCL, bandage contact lens. The experiment was conducted once using 20 A lenses and 20 B lenses.

Based on the Kaplan–Meier analysis, the A lenses had a significantly higher retention rate than the B lenses (*p* = 0.006, Figure 1). In total, 14/20 A lenses (70.0%, 95% confidence interval [95% CI]: 45.7%–88.1%) remained on the corneal surface for 168 h (7 days). This value was significantly higher than that obtained for the B lens retention, where 6/20 (30.0%, 95% CI: 11.9%–54.3%) lenses remained in place for 168 h (chi-squared test, *p* = 0.027). Within 24 h, 45.0% of the B lenses dropped, and 55.0% were lost within 48 h. Meanwhile, 90.0% of the A lenses remained attached after wearing them for 24 h, and 80.0% were retained after 48 h. The mean± standard deviation retention times of A and B lenses were 129.3 ± 61.7 and 66.1 ± 69.6 h, respectively.

**Figure 2:**
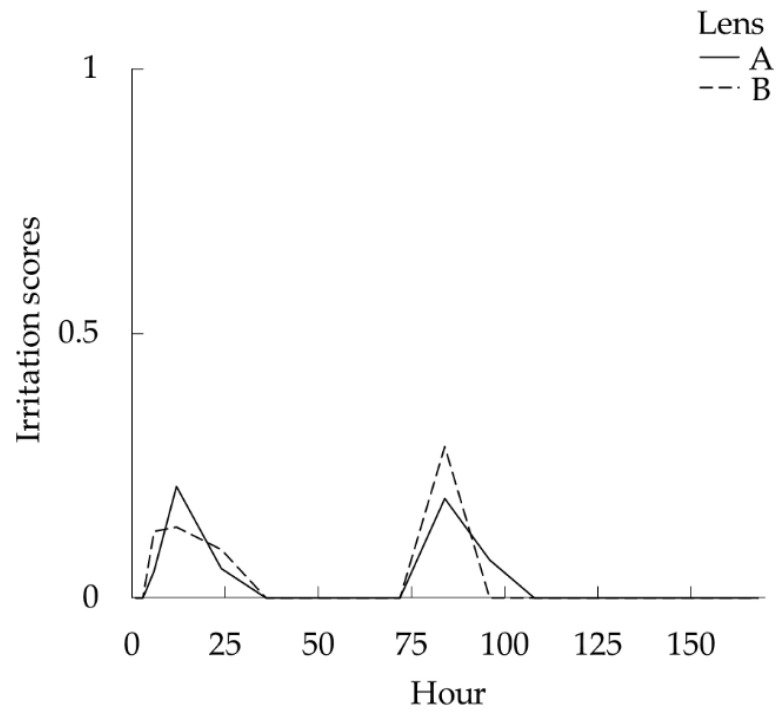
Changes on average irritation scores (ocular discharge) for A and B lenses.

As shown in Table 2 and Figure 2, there was no significant difference between the two types of BCLs in terms of irritation scores (*p* = 1). In total, 275 and 162 assessments were performed to evaluate the A and B lenses, respectively. Mild ocular discharge was noted in canines with the A lens based on 13 (4.7%)/275 recordings and canines with the B lens based on 7 (4.3%)/162 recordings. However, none of the eyes presented with ocular discomfort, conjunctival hyperemia, and fluorescein positivity.

### 3.2. Bacterial Isolation

Two strains of *S. pseudintermedius* (KS201 and KS202) were isolated from the conjunctival sac of two healthy canines. These *S. pseudintermedius* strains were used for the following in vitro studies.

### 3.3. In Vitro Bacterial Adhesion

**Figure 3:**
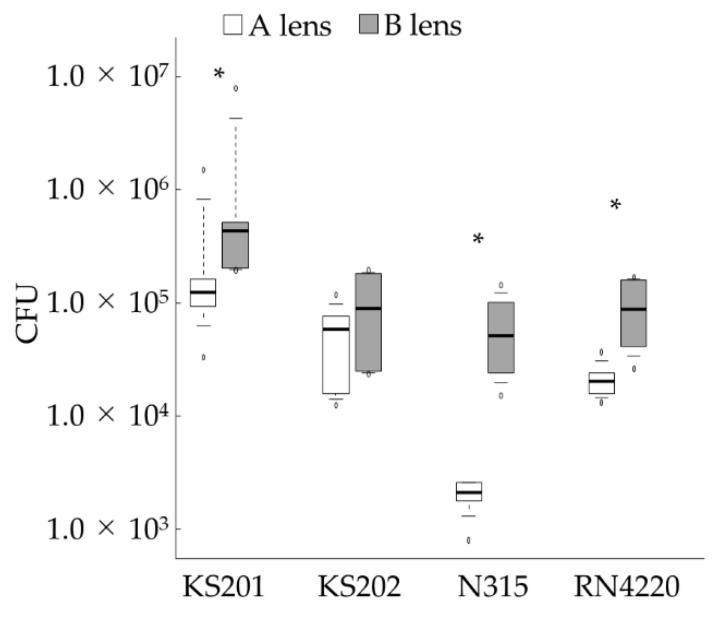
Number of bacteria adhering to the BCLs after 2 h of incubation. Data are presented as median (solid line), interquartile range (box), 10th–90th percentile (whiskers), and minimum and maximum (white circles). * *p* < 0.05. BCL, Bandage contact lens; CFU, colony forming unit. KS201 and KS202 are two strains of *Staphylococcus pseudintermedius*; N315 and RN4220 are two strains of *Staphylococcus aureus*. The experiment was conducted once using six A and six B lenses per bacterial strain.

To assess bacterial adhesion to BCLs, we counted the number of *Staphylococcus* spp. adhering to each BCL in vitro. Figure 3 shows the number of CFUs/lens. For all tested bacterial strains, with the exception of KS202 (*p* = 0.394), the B lens had significantly higher numbers of attached bacterial cells than the A lens (*p* = 0.041, KS201; *p* = 0.005, N315; *p* = 0.002, RN4220).

### 3.4. In Vitro Biofilm Formation

**Figure 4:**
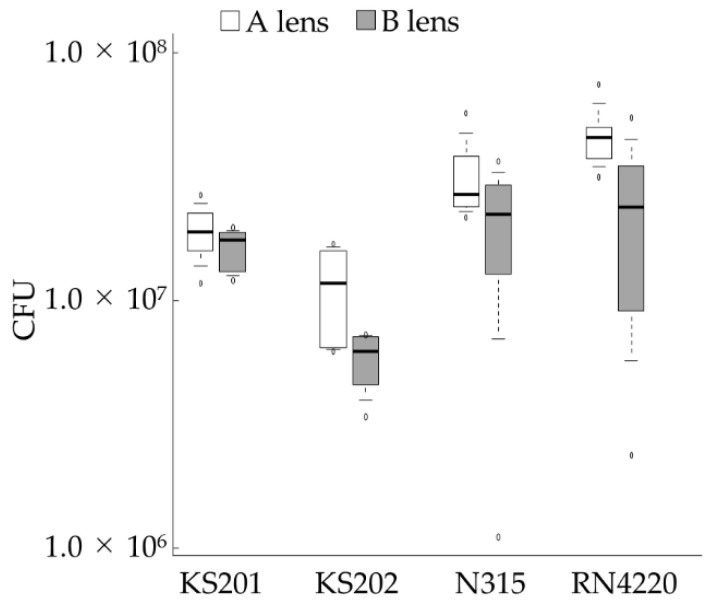
Number of bacteria obtained from the homogenized biofilm adhering to BCLs after 24 h of incubation. Data are presented as median (solid line), interquartile range (box), 10th–90th percentile (whiskers), and minimum and maximum (white circles). BCL, bandage contact lens; CFU, colony forming unit. The experiment was conducted once using six A and six B lenses per bacterial strain.

The number of *Staphylococcus* spp. in homogenized biofilms on each BCL was counted in vitro. Figure 4 depicts the number of CFUs/lens. Biofilm formation did not significantly differ between the two BCLs in KS201 and N315 (*p* = 0.589 and *p* = 0.240, respectively). However, in contrast to the results obtained in Section 3.3, it was more likely for the amount of biofilm formed on A lenses to be greater than that formed on the B lenses in KS202 and RN4220 (both *p* = 0.065).

### 3.5. In Vivo Bacterial Adhesion

To compare the bacterial adhesion on BCLs after wearing them for 24 h, we counted the number of bacteria adhering to each BCL in vivo. During the experiment, 17 A lenses and 11 B lenses were obtained. Table 3 shows the mean ± standard deviation of the CFUs isolated from both BCLs. The number of CFUs did not significantly differ (*p* = 0.549) in each BCL.

## 4. Discussion

This study revealed that the A lens was retained significantly better than the B lens. In the in vitro bacterial adhesion experiments, fewer CFUs were observed on the A lenses than on the B lenses for three out of four tested bacterial strains; however, more biofilm was formed on the A lenses in vitro than on the B lenses. Nevertheless, there was no significant difference between in vivo bacterial adhesion between the BCL types, and both BCLs were adequately tolerated by the intact canine ocular surface.

Previous studies have reported different BCL retention rates in canines [1,2,3,4,8,9]. Several factors, such as higher rigidity, greater thickness, and larger diameter may lower BCL retention rates [1,8]. Diehl et al. revealed that the oxygen permeability or water content of the BCL might affect the retention rate [9]. In our preliminary study, the ratio of the elastic modulus of the A lens to that of the B lens was 1.6:1. Therefore, the B lens is more flexible than the A lens. The finding that B lenses had lower retention rates is inconsistent with the abovementioned report showing that more flexible BCLs are superior in terms of retention. Because the BCLs used in this study were similar in terms of the BC, diameter, central thickness, and water content, the superiority of the A lens might be attributed to other factors such as ionicity and oxygen permeability.

In terms of in vitro bacterial adhesion and biofilm formation, the A lens had a significantly lower rate of bacterial adhesion in most strains tested, but was more likely to form a greater amount of biofilm than the B lens. In general, the initial step of biofilm formation is the adhesion of microorganisms to either biotic or abiotic surfaces [32,33]; however, recent studies have revealed that the trends for initial bacterial adhesion and biofilm formation later were not always similar [33]. For instance, Gottenbos et al. [21] reported that *S. aureus* had a slower initial adhesion to negatively charged materials than to positively charged materials. Meanwhile, exponential bacterial proliferation was only observed on negatively charged materials. Terada et al. [34]. reported that negatively charged *Escherichia coli* showed greater initial adhesion to positively charged material, but cell viability and biofilm growth were reduced in case of positively charged material than for negatively charged material. The zwitterionic B lens contains both negatively and positively charged groups, with an overall neutral charge. Therefore, theoretically, no electrostatic repulsion or attraction should occur between the lens and negatively charged bacteria, such as *Staphylococcus* spp. Our results showed that the anionic A lens allowed for less initial bacterial adhesion in vitro, potentially due to electrostatic repulsion, but did not inhibit bacterial proliferation and even promoted biofilm formation compared to the B lens. The conflicting findings of the bacterial adhesion and biofilm formation in vitro experiment may be attributed to cell viability.

This study did not show significant differences in terms of in vivo bacterial adhesion between the two types of BCLs after short-term wear (24 h). The negatively charged BCL rapidly takes up lacrimal proteins, which create a suitable environment for microorganisms [13,14,15,18]. Among the lacrimal proteins, lysozyme, which is a positively charged antimicrobial enzyme, has been detected in high concentrations on BCLs and may be beneficial for preventing microbial adhesion during lens wear [14,18]. This antimicrobial effect may be associated with the lack of significant in vivo bacterial adhesion observed on the A lens.

In this study, the biofilm formed on the BCL was quantified by counting the number of bacteria in the homogenized biofilm. The spectrophotometric method using crystal violet is the most common method for quantifying biofilms [29,30,35,36,37,38]. We initially used the crystal violet biofilm quantification method; however, this method was inappropriate due to the hydrophilicity of the BCL and the absorption of crystal violet. Additionally, Kim et al. [16] demonstrated that the crystal violet method is not effective. Thus, we chose an indirect quantification method to quantify the biofilm, which involves counting the number of bacteria obtained from the homogenized biofilm adhering to the BCLs. This approach has become the preferred alternative to the crystal violet method, and several studies have quantified biofilm development on intraocular lenses and BCLs via indirect quantification [13,16,29,30].

This study had several limitations. First, we used two differently charged BCLs with a similar BC, diameter, central thickness, and water content. However, some properties such as edge design, roughness, and hydrophilicity were not considered. The edge design of a BCL is important in fitting and comfort [39], and BCL surface roughness and hydrophilicity are correlated with bacterial adhesion [28]. Second, we used healthy canines in our in vivo studies. A recent study showed that canine eyes with various ophthalmic diseases, such as corneal ulceration, uveitis, and glaucoma, had a higher lacrimal albumin levels than healthy eyes [40]. Albumin is negatively charged, and the deposition of albumin on BCLs enhances bacterial adhesion [14]. Therefore, bacterial adhesion to BCLs may differ between healthy eyes and those with ocular diseases. Third, the 24 h test period for the in vivo bacterial adhesion is short because canine BCLs are typically placed for a few weeks. We initially planned to evaluate bacterial adhesion on BCLs worn for 7 days; however, due to poor retention of B lenses after 7 days, it was difficult to obtain a sufficient number of samples. Thus, we compared in vivo bacterial adhesion between A and B lenses worn for 24 h. Fourth, negative controls were not included in the in vivo bacterial adhesion study. During BCL placement and removal, sterilized soft-tip forceps were used, and caution was taken to avoid the BCL from coming into contact with the canine’s eyelids and eyelashes so as to prevent the BCLs from being contaminated by the flora present on the experimenter’s fingers and canine’s eyelids and eyelashes. However, there was a possibility that airborne bacteria could have contaminated the samples.

## 5. Conclusions

Both lenses were well tolerated and the anionic BCL were superior to the more flexible zwitterionic BCL in term of retention but not biofilm formation in vitro. The overall in vivo bacterial adhesion did not show significantly difference between two types of BCLs after short-term wearing. Thus, their short-term use in dogs can be recommended as safe. Nevertheless, further studies considering the edge design, roughness, and hydrophilicity of BCLs and evaluating long-term use must be performed.

## Figures and Tables

**Table 1:** Overview of key criteria of both BCLs.

	A Lens	B Lens
Material	Etafilcon A	SIB
Ionicity	Anionic	Zwitterionic
BC (mm)	8.9/9.3	8.9/9.3
DIA (mm)	16.0	16.0
CT (mm)	0.25	0.25
WC (%)	58	58

BCL, bandage contact lens; SIB, SEED ionic bond; BC, curvature of lens; DIA, diameter; CT, central thickness; WC, water content.

**Table 2:** Irritation scores (ocular discharge) for the A and B lenses.

Grade	Both Lenses	A Lens	B Lens
0	417 (95.4%)	262 (95.3%)	155 (95.7%)
1	020 (4.6%)	013 (4.7%)	007 (4.3%)
2	000 (0%)	000 (0%)	000 (0%)
3	000 (0%)	000 (0%)	000 (0%)
Total recordings	437	275	162

The irritation scores of both lenses were low (grades 0–1), which was common to both groups, and there was no significant difference between the two types of lenses worn in terms of irritation scores (*p* = 1). Discomfort, conjunctival hyperemia, and fluorescein uptake were not observed (scored 0 for both lenses).

**Table 3:** Number of bacteria adhering to the BCLs after continuously wearing them for 24 h.

	A Lens	B Lens
CFU/lens	35.9 ± 38.4	49.1 ± 91.4

Data are presented as mean ± standard deviation. BCL, bandage contact lens; CFU, colony forming unit.

## Data Availability

The data presented in this study are available upon request from the corresponding author.
